# Acromelic dysplasias: similarities and differences in clinical and molecular findings in 12 Turkish patients

**DOI:** 10.1007/s00431-026-07075-2

**Published:** 2026-05-19

**Authors:** N. Güneş, S. Türk, H. Onur, K. Gür, C. Yüksel Elgin, E. Çifçi Sunamak, D. Uludağ Alkaya, A. Güler Eroğlu, B. Tüysüz

**Affiliations:** 1 Department of Pediatric Genetics, Cerrahpaşa Faculty of Medicine, Istanbul University-Cerrahpaşa, Istanbul, Turkey; 2 Department of Ophthalmology, Cerrahpaşa Faculty of Medicine, Istanbul University-Cerrahpaşa, Istanbul, Turkey; 3 Department of Pediatric Cardiology, Cerrahpaşa Faculty of Medicine, Istanbul University-Cerrahpaşa, Istanbul, Turkey; 4 Department of Pediatrics, Faculty of Medicine, Istanbul Atlas University, Istanbul, Turkey

**Keywords:** Acromelic dysplasia, *FBN1*, *ADAMTSL2*, *ADAMTS10*, *ADAMTS17*, *GNAS*

## Abstract

The purpose of this study is to compare the natural history of clinical and radiologic features in patients with acromelic dysplasias. Twelve patients from nine families with genetically confirmed dysplasia types with acromelia were included in the study, and eight of them were followed-up for a median of 8.1 years. Monoallelic disease-causing variants were identified in *FBN1* (acromicric dysplasia, *n* = 3) and *GNAS* (Albright hereditary osteodystrophy (AHO), *n* = 1). Biallelic disease-causing variants in *ADAMTSL2* (geleophysic dysplasia type 1, *n* = 4), *ADAMTS10* (Weill–Marchesani syndrome type 1 (WMS1), *n* = 3), and *ADAMTS17* (Weill–Marchesani syndrome type 4 (WMS4), *n* = 1) were identified. Five novel variants were detected. Short stature was present in all patients. In all patients with geleophysic dysplasia, height normalized during follow-up, and in two, initial acromelia and broad phalanges on hand radiographs resolved over time. Pseudomuscular build, joint limitations, tiptoe walking, and delayed bone age were common findings in geleophysic dysplasia, while patients with WMS1 also had pseudomuscular build, joint limitations, and delayed bone age. Acromicric dysplasia showed mild joint limitation. Intellectual disability was observed only in the WMS4 patient. Spherophakia was specific to patients with WMS. Heterotopic ossification was present in the AHO patient.

*Conclusion*: These findings underscore the clinical and genetic heterogeneity of acromelic dysplasias and emphasize that integrated clinical and molecular evaluation is essential for accurate classification and follow-up.

**Table:** 

**What is known:** • *Acromelic dysplasias are rare connective tissue disorders characterized by short stature, brachydactyly, and joint stiffness, caused by variants in genes involved in extracellular matrix organization and TGF-β– related signaling.*	
**What is new:** • *Five novel variants in ADAMTSL2, ADAMTS10, and ADAMTS17 expand the molecular spectrum of acromelic phenotypes.* • *Tiptoe walking, in association with early findings including short stature, acromelia, and broad proximal phalanges on radiographs, may suggest ADAMTSL2-related geleophysic dysplasia.*

**What is known:**

• *Acromelic dysplasias are rare connective tissue disorders characterized by short stature, brachydactyly, and joint stiffness, caused by variants in genes involved in extracellular matrix organization and TGF-β– related signaling.*

**What is new:**

• *Five novel variants in ADAMTSL2, ADAMTS10, and ADAMTS17 expand the molecular spectrum of acromelic phenotypes.*

• *Tiptoe walking, in association with early findings including short stature, acromelia, and broad proximal phalanges on radiographs, may suggest ADAMTSL2-related geleophysic dysplasia.*

## Introduction

Acromelic dysplasias are rare congenital connective tissue disorders characterized by short stature, brachydactyly, progressive joint stiffness, pseudomuscular build, and multisystem involvement affecting the eyes, heart, and respiratory tract [1, 2]. These disorders are caused by defects in proteins of the microfibrillar network, a key component of the extracellular matrix that regulates both tissue mechanics and the bioavailability and signaling activity of the transforming growth factor beta (TGF-β) superfamily [1–3]. The four individual disorders belonging to the acromelic dysplasia group are geleophysic dysplasia, acromicric dysplasia, Weill–Marchesani syndrome (WMS), and Albright hereditary osteodystrophy (AHO) [3].

Geleophysic dysplasia results from biallelic pathogenic variants in *ADAMTSL2* (Geleophysic dysplasia type 1, MIM #231050), monoallelic pathogenic variants in *FBN1* (Geleophysic dysplasia type 2, MIM #614185) and *LTBP3* (Geleophysic dysplasia type 3, MIM #617809) [1]. In addition, monoallelic pathogenic variants in *FBN1* is also implicated in two other acromelic dysplasias: acromicric dysplasia (MIM #102370) and WMS2 (MIM #608328). The recessive forms of WMS include WMS1 (MIM #277600), WMS3 (MIM #614819), and WMS4 (MIM #613195), which are caused by biallelic pathogenic variants in *ADAMTS10*, *LTBP2*, and *ADAMTS17*, respectively [1, 2]. Albright hereditary osteodystrophy (MIM #103580) results from heterozygous pathogenic variants in *GNAS* [4].

This study aimed to comprehensively describe the clinical and radiographic spectrum of acromelic dysplasias and to further delineate genotype–phenotype correlations.

## Materials and methods

### Patients

The study included a total of 12 patients (P) from 10 unrelated families (F) diagnosed with dysplasia syndromes presenting with acromelia who were retrospectively identified from patients evaluated and followed at a single tertiary referral center. Only individuals with a molecularly confirmed diagnosis were included.

Based on detailed clinical evaluation, patients were diagnosed with geleophysic dysplasia, acromicric dysplasia, WMS, and Albright hereditary osteodystrophy. Diagnoses were established according to previously defined clinical criteria [5–7].

Written informed consent was obtained from participants or their legal guardians in compliance with international ethical standards and the Declaration of Helsinki. The consent covered both molecular analysis and the publication of clinical and molecular data, including photographs. This study was approved by the local ethics committee.

### Molecular genetic analysis

Genomic DNA was isolated from peripheral blood using the salting-out method. Exome sequencing was performed with the QIAseq Human Exome Kit (Qiagen GmbH, Hilden, Germany), and reads were aligned to GRCh37/hg19 using BWA-MEM (https://github.com/lh3/bwa). Variant detection was performed with the GATK HaplotypeCaller tool (https://gatk.broadinstitute.org/hc/en-us), and annotation was done via ANNOVAR (https://annovar.openbioinformatics.org/en/latest). Variants with a genotype quality score of ≥ 20 and a minor allele frequency of ≤ 1% in gnomAD were included. Pathogenicity was evaluated using in silico prediction tools (PolyPhen-2, SIFT, MutationTaster, DANN) and databases (dbSNP, ExAC, 1000 Genomes, ClinVar, VarSome, HGMD Professional version). Variants were interpreted according to the American College of Medical Genetics and Genomics (ACMG) guidelines [8]. All novel variants were confirmed by Sanger sequencing, and segregation analysis was performed.

## Results

### Baseline characteristics

The cohort included six males and eight females. Median age at first presentation was 6.5 (range, 0.2–24.6) years. Patients were followed for a median of 8.1 (range, 1.6–16.5) years.

**Fig. 1:**
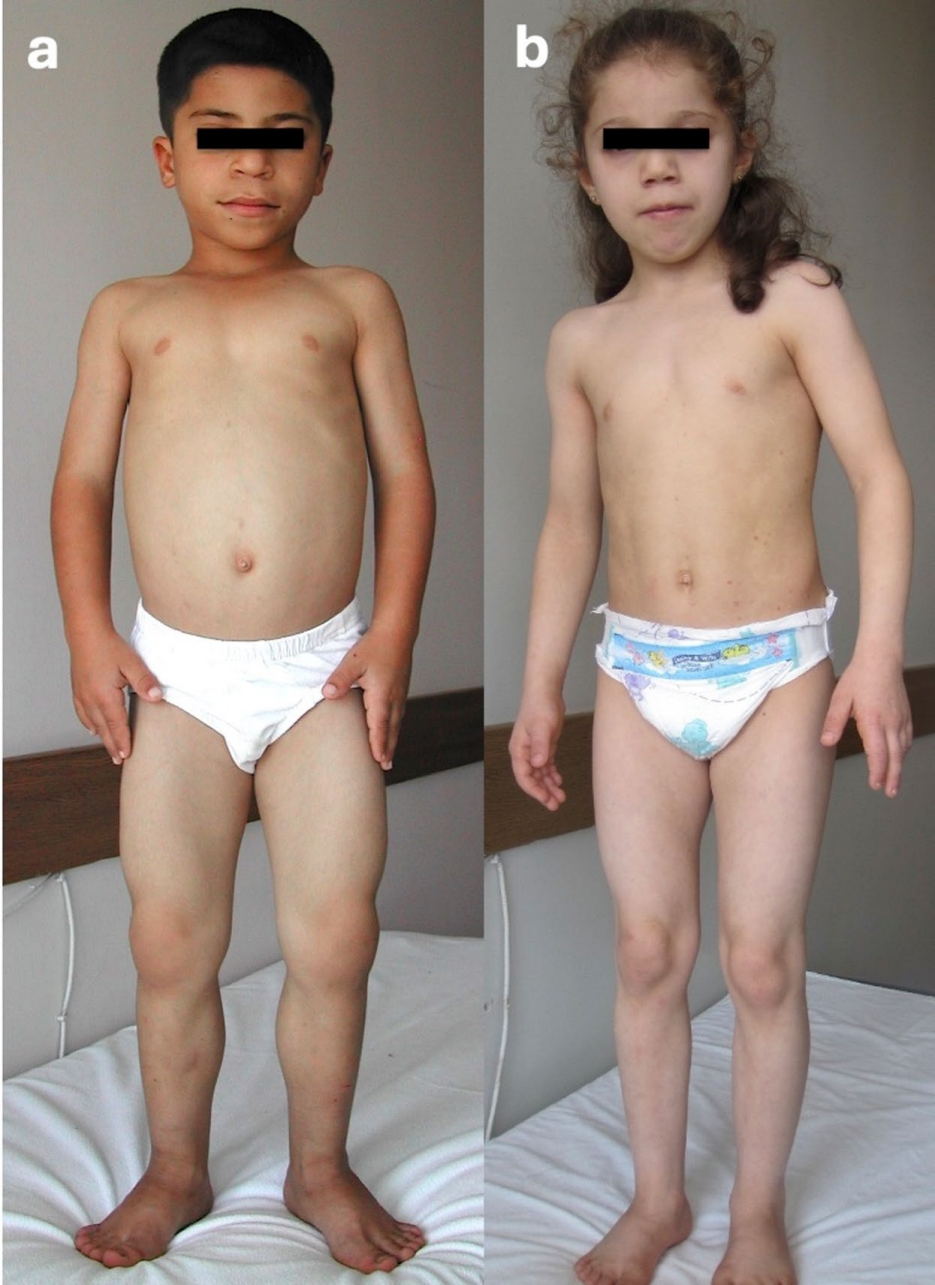
Photographs of two patients (**a** and** b**). P1 with geleophysic dysplasia exhibited upslanting palpebral fissures, right-sided ptosis, long smooth philtrum, wide mouth, and a pseudomuscular build at 8.3 years of age (**a**). P11 with Weill–Marchesani syndrome type 4 had facial features including a long and smooth philtrum, thin vermilion of the lips, and prognathism at 10.7 years (**b**)

**Fig. 2:**
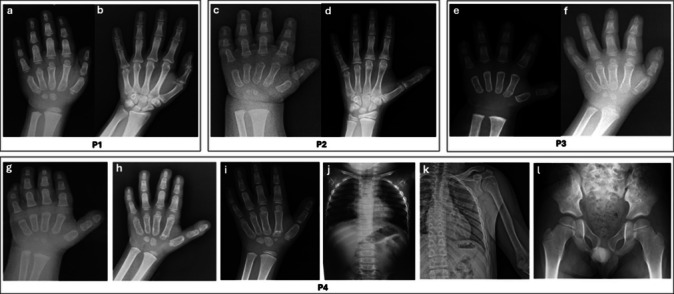
Hand radiographs of four patients (P1–P4) with geleophysic dysplasia (**a**–**i**), posteroanterior chest radiographs (**j** and **k**), and a pelvic radiograph (**l**) of P4. P1 had delayed carpal ossification, short fourth and fifth metacarpal bones, broad proximal phalanges, and short middle and distal phalanges at 7.6 years of age (**a**); these findings were less pronounced at 24.1 years (**b**). P2 exhibited very small carpal bones, short third–fifth metacarpal bones with rounded proximal ends, and short phalanges at 1.8 years (**c**); however, on the follow-up hand radiograph at 13.7 years (**d**), these findings were less pronounced. P3 had non-ossified carpal bones, short tubular bones, and broad proximal phalanges at 1.5 years (**e**); shortening of tubular bones and delayed bone age persisted at 5.1 years (**f**). In addition to short tubular bones and broad proximal phalanges, P4 showed shortening of the fifth middle phalanx at 0.2 (**g**), 4.7 (**h**), and 7.2 (**i**) years. P4 also had rib widening that became more pronounced with age, observed at 0.2 (**j**), and 16.5 (**k**) years; whereas the pelvic radiograph of P4 showed coxa valga and short femoral necks at 7.2 years of age (**l**)

**Fig. 3:**
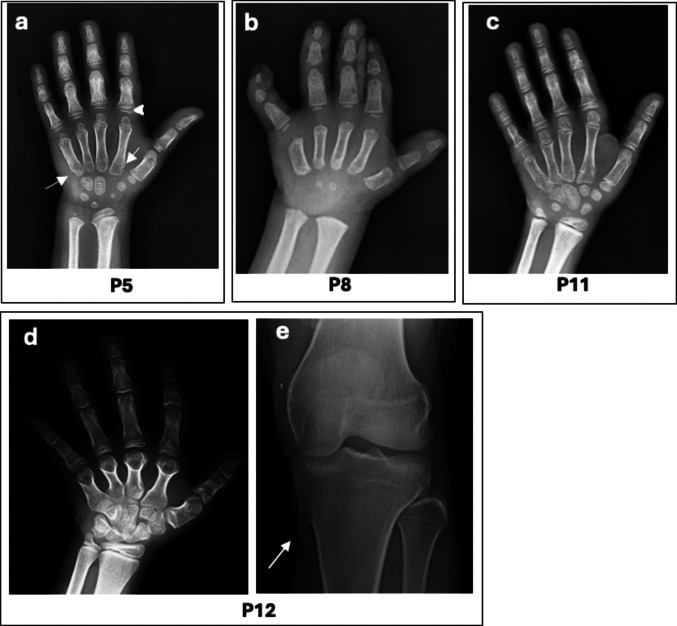
The radiograph of P5 with acromicric dysplasia at 5.4 years showed cone-shaped epiphyses (arrowhead), notching of the second and fifth metacarpals (arrows), short and stubby proximal and middle phalanges, and delayed bone age (**a**). Radiographs of P8 with Weill–Marchesani syndrome (WMS) type 1 at 3 years of age (**b**) and P11 with WMS type 4 at 10.8 years of age (**c**) showed short tubular bones and marked shortening of the fifth middle phalanges. P8 also had delayed bone age, broad and short proximal phalanges, and a broad fifth metacarpal bone (**b**). The hand radiograph of P12 with Albright hereditary osteodystrophy at 8.6 years of age showed short and broad metacarpal bones (**d**), and heterotopic ossification in the medial region of the left tibia (arrow) was observed at 15.2 years of age (**e**)

Molecular and clinical data are summarized in Table 1, while patient photographs and radiographs are shown in Figs. 1, 2, and 3.

**Table 1:** Clinical and molecular characteristics of the patient cohort according to the dysplasia subtype

Clinical phenotype	Geleophysic dysplasia				Acromicric dysplasia			Weill–Marchesani syndrome				Albright hereditary osteodystrophy
Patient number	1	2	3	4	5	6	7	8	9	10	11	12
Gender	M	M	M	M	F	F	M	M	F	M	F	F
Gene	*ADAMTSL2*		*ADAMTSL2*	*ADAMTSL2*	*FBN1*	*FBN1*		*ADAMTS10*	*ADAMTS10*		*ADAMTS17*	*GNAS*
Detected variant	c.286C > T, p.Arg96Trp (hom.)		c.2053 T > A, p.Cys685Ser (hom.)	c.1400C > G, p.Ser467Cys (hom.)	c.5096A > G, p.Tyr1699Cys (het.)	c.5284G > A, p.Gly1762Ser (het.)		c.1191-1G > T, p.? (hom.)	c.1040G > A, p.Arg347His (hom.)		c.1118G > A, p.Cys373Tyr (hom.)	c.529 T > A, p.Tyr177Asn (het.)
Novelty/ACMG classification (evidence)	Known (ref. 9)/VUS (PM2)		Novel/VUS (PM2, PP3)	Novel/VUS (PM2)	Known (ref. 11–15)/P (PS2, PM1, PP2, PM2, PM5, PP3)	Known (ref. 18)/P (PS2, PM2, PM5, PP2, PP5)		Novel/LP (PVS1, PM2)	Novel/LP (PP3, PM2, BP1)		Novel/VUS (PM2, PP3)	Known/VUS (PM1, PP2, PM2, PP3)
Referral indication	Short stature, tiptoe walking	Short stature	Dysmorphic facies	Dysmorphic facies, brachydactyly	Short stature	Short stature	Short stature	Brachydactyly	Congenital heart disease	Congenital heart disease	Microspherophakia, iridodonesis	Brachydactyly
Age (yr.) at admission/last visit	7.6/24.1	1.8/13.7	1.5/5.1	0.2/9.7	5.4/7.0	24.6/-	1.2/-	0.2/14.2	12.3/-	8.7/-	10.7/16.7	8.5/15.2
Height (SDS) at admission/last visit	− 2.8/− 1.8	− 3.0/− 1.8	− 3.2/− 1.8	− 2.1/− 1.9	− 5.5/− 6.8	− 5.7/	− 3.2/−	− 2.5/− 4.3	− 4.5/−	− 3.0/−	− 4.1/− 4.0	− 1.3/− 3.1
Hand length at admission/last visit	< 3p./25–50p.	< 3p./3–25p.	< 3p./3p.	< 3p./< 3p.	< 3p./< 3p.	< 3p./-	< 3p./-	< 3p./< 3p.	< 3p./-	< 3p./-	< 3p./< 3p.	< 3p./< 3p.
Pseudomuscular build	+	+	−	−	−	−	−	+	+ (mild)	+ (moderate)	−	−
Joint limitations	+	+	+	+	+	−	−	+	+	+	−	−
Tiptoe walking	+	+	+	+	−	−	NA	−	−	+	−	−
Spherophakia	−	−	−	−	−	−	−	+	+	+	+	−
Ectopia lentis	−	−	−	−	−	−	−	+	+	+	−	−
Glaucoma	−	−	−	−	−	−	−	+	+	+	−	−
Myopia	−	−	−	+	−	−	−	−	+	+	−	−
Strabismus	−	−	−	+	−	−	−	−	−	−	−	−
Hepatomegaly	+	−	+	−	+	−	+	−	−	−	−	−
Cardiac involvement	ASD	ASD	MR (mild)	PS	MR	NA	NA	MR (mild)	AS (op.)	PS (op.), AS (mild)	−	NA
Intellectual disability	−	−	−	−	−	−	−	−	–	–	+ (IQ = 45)	−
X-ray findings of the hand												
Short tubular bones	+	+	+	+	+	NA	+	+	+	+	+	+
Broad phalanges	+	+	+	+	+		+	+	+	+	−	−
Short fifth middle phalanx	−	−	−	+	−		−	+	+	+	+	−
Delayed bone age	+	+	+	+	+		−	+	+	+	+	−
Other features	Achilloplasty (op.), ptosis	Achilloplasty (op.), ptosis		Achilloplasty (op.)	Wide 1. and 5. metacarpals	Epilepsy (under medical treatment)	Wide 1. and 5. metacarpals	Glaucoma (op.), astigmatism, hydrocele				Heterotopic ossification

*ACMG* American College of Medical Genetics, *AS* aortic stenosis, *ASD* atrial septal defect, *F* female, *LP* likely pathogenic, *M* male, *MR* mitral regurgitation, *NA* not available, *op*. operated, *P* pathogenic, *p*. percentile, *PS* pulmonary stenosis, *Ref.* reference, *SDS* standard deviation score, *VUS* variant of uncertain significance, *yr.* years

### Clinical findings

Patients were classified according to the clinical criteria described in the Methods section; four patients were compatible with geleophysic dysplasia type 1, three with acromicric dysplasia, three with WMS1, one with WMS4, and one with AHO.

#### Patients diagnosed with geleophysic dysplasia

P1–4 were diagnosed with geleophysic dysplasia, all of whom were male. The main referral indications were dysmorphic facial features including hypertelorism, epicanthal folds and long philtrum in two patients, and short stature in two further patients, one of whom was also referred because of brachydactyly.

At admission, ages ranged from 0.2 to 7.6 years, and at the last visit from 5.1 to 24.1 years. Height standard deviation score (SDS) at the first visit ranged between − 2.1 and − 3.2, improved to a range of − 1.8 to − 1.9 at the last visit. While hand length was below the 3rd percentile in all patients at admission, with partial improvement in two cases at follow-up.

All four patients had distinctive facial features including hypertelorism, broad nasal bridge, long flat philtrum, and thin vermilion of the upper lip. P1 and P2 had unilateral ptosis (Fig. 1a). Joint limitations were observed in all patients, mainly affecting large joints. All patients exhibited tiptoe walking. Three patients underwent Achilles’ tendon surgery. Pseudomuscular build was observed in two patients. Cardiac involvement included an atrial septal defect in two patients, mild mitral regurgitation in one patient, and pulmonary stenosis in one patient. None had intellectual disability.

Radiographic evaluation of the hands (Fig. 2a–i) consistently showed short tubular bones and broad proximal phalanges. These findings appeared less pronounced at follow-up in two patients (P1, P2). Delayed bone age was present in four patients. Shortening of the fifth middle phalanx was additionally observed in P4. P4 showed coxa valga and short femoral necks (Fig. 2l), and increased rib width (Fig. 2k) at follow-up.

#### Patients diagnosed with acromicric dysplasia

P5–7 were diagnosed with acromicric dysplasia, including one female (P5) from one family and one female (P6) and male (P7) from another family. All presented with short stature as the main referral indication.

At admission, height SDS ranged from − 3.2 to − 5.7. In one patient (P5) with follow-up data, height SDS decreased from − 5.5 to − 6.8. Hand length was below the 3rd percentile in all patients.

All patients exhibited distinct facial features including bulbous nose with anteverted nostrils, long and prominent philtrum, and thick lips with a small mouth. One patient (P5) had joint limitations. Cardiac involvement included mild mitral regurgitation and mitral valve thickening in one patient (P5), while data were unavailable for two patients. No ophthalmologic abnormalities were detected in three patients. Epilepsy under medical treatment was reported in one adult patient (P6).

Hand radiographs of P5 and P7 revealed short tubular bones with broad proximal and middle phalanges. Additionally, they had widened the first and fifth metacarpals, notching the second and fifth metacarpals. Delayed bone age was present only in P5 (Fig. 3a).

#### Patients diagnosed with Weill–Marchesani syndrome

P8–10 were diagnosed with WMS1, P9 and P10 were siblings, and P11 was diagnosed with WMS4.

At the first examination, height SDSs were − 2.5 and − 4.5. Hand length was below the 3rd percentile in all patients. Facial features, including a flat philtrum and a thin vermillion of the lips, were observed in P9 and P10 with WMS1, as well as in P11 with WMS4 (Fig. 1b). A pseudomuscular build was observed in all WMS1 patients (P8, P9, and P10), with variable severity: it was pronounced in P10, one of the siblings, while it was present in a milder form in P9. This feature was not observed in the WMS4 patient (P11). Joint limitation was present in all patients with WMS1, whereas it was not detected in the WMS4 patient. Tiptoe walking was noted only in P10, one of the siblings with WMS1.

All patients showed ophthalmologic involvement; P8 presented with spherophakia and ectopia lentis and required surgery for glaucoma at 7.9 years of age. Similarly, the siblings with WMS1 (P9, P10) also had spherophakia, ectopia lentis, and glaucoma. P11 with WMS4 presented only with spherophakia.

Mild mitral regurgitation was present in P8 with WMS1. Among the siblings with WMS1, P9 underwent surgery at 3 years of age due to aortic stenosis, whereas P10 was operated on at 9 months of age for pulmonary stenosis. Echocardiographic evaluation of P11 with WMS4 was normal. Intellectual disability (IQ = 45) was observed in P11, whereas other patients had normal cognitive development.

Hand radiographs of all patients revealed short tubular bones, shortening of the fifth middle phalanx, and delayed bone age (Fig. 3b and c). P8, P9, and P10 also had broad proximal and middle phalanges (Fig. 3b).

#### Patients diagnosed with Albright hereditary osteodystrophy

P12 was diagnosed with AHO. Her referral indication was brachydactyly.

At the initial evaluation at 8.5 years of age, her height SDS was − 1.3; however, at the last follow-up her height SDS had decreased to below − 2. Hand length was below the 3rd percentile, and neurodevelopment was normal. Biochemically, she had persistently elevated parathyroid hormone (PTH) levels with normal serum calcium and phosphate concentrations, and thyroid function tests were within the normal range.

Radiographs revealed short and wide metacarpal bones (Fig. 3d) and heterotopic ossification (Fig. 3e).

### Molecular findings

Genetic analysis identified VUS (5/9), pathogenic (2/9), and likely pathogenic (2/9) variants. Variants were detected in the following genes: *ADAMTSL2* (NM_014694.4) in three families with geleophysic dysplasia type 1; *FBN1* (NM_000138.5) in two families diagnosed with Acromicric dysplasia; *ADAMTS10* (NM_030957.4) in three WMS1 patients*; ADAMTS17* (NM_039057.4) in one patient diagnosed with WMS4; *GNAS* (NM_000516.7) in one AHO patient (Table 1).

All variants were missense variants, except for the splice-site variant identified in *ADAMTS10*. Variants in *FBN1* and *GNAS* were heterozygous, whereas variants in *ADAMTSL2*, *ADAMTS10*, and *ADAMTS17* were homozygous.

Five novel variants were identified: two variants in *ADAMTSL2* (c.2053T > A, c.1400C > G), two variants in *ADAMTS10* (c.1040G > A, c.1191-1G > T), and one variant in *ADAMTS17* (c.1118G > A). Among these variants, three were classified as VUS, while two were categorized as likely pathogenic.

## Discussion

Acromelic dysplasias share a distinctive pattern of multisystem involvement that primarily affects the musculoskeletal system, skin, eyes, and, in some subtypes, the cardiovascular and respiratory systems [2]. The core clinical features include proportionate short stature, brachydactyly, progressive joint stiffness, and a characteristic pseudomuscular body habitus [1]. These disorders result from pathogenic variants in genes encoding secreted extracellular matrix proteins—such as *FBN1*, *ADAMTSL2*, *ADAMTS10*, *ADAMTS17*, *LTBP2*, and *LTBP3*—as well as in *SMAD4* [1, 2]. The overlapping skeletal and connective tissue features suggest that these proteins function within a shared biological pathway, while also exerting tissue-specific effects that account for phenotypic variability among different acromelic dysplasia subtypes [2].

*ADAMTSL2* encodes an extracellular matrix glycoprotein essential for microfibrillar organization, and its loss disrupts interactions with fibrillin-1 and fibrillin-2 [9]. Studies have reported largely overlapping clinical features between *ADAMTSL2*-related and *FBN1*-related geleophysic dysplasia, whereas subtle differences—such as facial dysmorphism (thin upper lip, long flat philtrum, and narrow palpebral fissures) and tiptoe walking—have been described to be more consistent in *ADAMTSL2*-related cases [10]. All four geleophysic dysplasia patients carrying *ADAMTSL2* variants in our cohort exhibited facial features including hypertelorism, broad nasal bridge, long flat philtrum, and thin upper lip, as well as tiptoe walking. The c.286C > T variant in *ADAMTSL2* has been reported in compound heterozygosity in an individual with geleophysic dysplasia [9]. This patient had severe short stature, joint contractures, tiptoe gait, scoliosis, and ocular abnormalities including bilateral ptosis, glaucoma, and corneal ectasia. The siblings (P1, P2) in our study with the same variant biallelically exhibited a milder phenotype with short stature, joint contractures, tiptoe walking, and isolated ptosis. Radiographs showed short tubular bones and broad proximal phalanges, both appearing less pronounced at follow-up. Two additional patients carrying novel *ADAMTSL2* variants (P3, P4) had short stature, brachydactyly, joint contractures, and tiptoe walking, without ptosis. Early radiographs in both showed broad proximal phalanges, while follow-up imaging in P4 revealed increased rib width. Delayed bone age, a known feature of the disease [11], was observed in all four patients. Overall, together with tiptoe walking and facial features, the combination of short stature, acromelia, and broad proximal phalanges on radiographs—particularly when recognized early—may serve as a potential clinical indicator for *ADAMTSL2*-related geleophysic dysplasia.

*FBN1* encodes fibrillin-1, a major component of extracellular microfibrils that maintain connective tissue integrity and regulate TGF-β bioavailability [12, 13]. The TGF-β-binding protein-like domain 5 (TB5) is critical for protein function: in-frame missense variants in this region are associated with acromelic dysplasias, whereas truncating variants, typically resulting in loss of function, are associated with Marfan syndrome [13]. The c.5096A > G; p.(Tyr1699Cys) variant in the TB5 domain has been identified in both geleophysic and acromicric dysplasia and may be associated with severe cardiopulmonary involvement, particularly in cysteine-altering TB5 variants [11–15]. Consistently, our patient (P5) with acromicric dysplasia and carrying this variant exhibited mitral valve involvement in addition to the characteristic features of acromicric dysplasia, including short stature with brachydactyly and distinctive facial features. The c.5284G > A variant, also affecting the TB5 domain, has been reported in both acromicric and geleophysic dysplasia with variable phenotypic expression, ranging from isolated short stature to multisystem involvement including cardiac, respiratory, and hepatic features [11, 15–18]. In our study, this variant was identified in a mother and her son with acromicric dysplasia, both presenting with short stature and brachydactyly, with additional hepatomegaly in the son. Overall, c.5284G > A represents a recurrent TB5 missense variant underlying acromelic dysplasia with variable phenotypic expression.

Clinically, WMS is characterized by ocular abnormalities—including ectopia lentis, microspherophakia, and cataract—along with musculoskeletal features of acromelic dysplasias [2]. The interaction of *ADAMTS10*, *ADAMTS17*, and *LTBP2* with fibrillin-1 in the ciliary zonule explains the occurrence of ectopia lentis. Cardiac valve abnormalities have frequently been described [2, 6]. Nevertheless, WMS4 was previously described as a WMS-like syndrome due to the relative absence of joint stiffness and cardiac abnormalities [2]. Consistently, joint limitations were significantly less frequent in WMS4 patients (2/9, 22%) compared with the other three WMS types (20/24, 83.3%) in a series [6]. Valvulopathy was also less common but not significantly different in WMS4 (2/12, 17% vs. 7/24, 29.1%). Similarly, patients with WMS1 (P8–10) and WMS4 (P11) in our study exhibited short stature, brachydactyly, and spherophakia; however, joint stiffness and cardiac involvement were observed only in WMS1 patients.

*GNAS* encodes the G protein α-subunit (Gsα), which couples seven-transmembrane receptors to adenylate cyclase, thereby regulating intracellular cAMP production in response to multiple hormones [4]. P12, carrying the heterozygous c.529T > A variant in *GNAS*, exhibited brachydactyly with short, broad metacarpal bones and heterotopic ossification; short stature developed during follow-up. She had persistently elevated PTH levels with normal serum calcium and phosphate concentrations. Another patient with a different *GNAS* variant (c.128T > C) and TSH resistance, characterized by elevated TSH with normal free thyroid hormone levels, was previously reported by our group [7]. However, thyroid function tests were within the normal range in the present case.

*SMAD4* encodes a central intracellular mediator of TGF-β and bone morphogenetic protein signaling, acting as a transcriptional coregulator that forms complexes with receptor-regulated SMADs [19]. Myhre syndrome is caused by heterozygous *SMAD4* variants, most commonly the recurrent c.1498A > G p.(Ile500Val) substitution [20–23]. In addition to short stature, characteristic facies, skeletal anomalies, joint contractures, and muscular build, patients carrying this variant exhibited neurodevelopmental delay, hearing loss, precocious puberty, and aortic coarctation [22]. In our previous report, a family carrying this variant showed intrafamilial variability, including intellectual disability, precocious puberty, aortic coarctation, hearing impairment, and early death due to recurrent infections [24].

Table 2 highlights that the distinguishing clinical features among acromelic dysplasia subtypes remain valuable for differential diagnosis despite overlapping phenotypes. Geleophysic dysplasia patients in our cohort exhibited joint stiffness, tiptoe walking, and delayed bone age. Short stature, acromelia, and broad proximal phalanges on hand radiographs were more prominent in early evaluations. These findings are concordant with prior large cohorts implicating dysregulated extracellular matrix homeostasis and altered TGF-β signaling as central mechanisms underlying progressive connective tissue fibrosis and skeletal restriction [11]. In contrast, individuals with acromicric dysplasia demonstrated milder joint involvement and characteristic notching of the second and fifth metacarpals, aligning with previous genotype–phenotype observations [5, 11]. Our WMS patients showed substantial phenotypic overlap with geleophysic dysplasia, particularly regarding brachydactyly and broad phalanges; however, anterior segment ocular abnormalities emerged as a key discriminative feature. This supports existing evidence from larger cohorts that *ADAMTS10* and *ADAMTS17*-related pathways critically affect zonular fiber integrity and anterior segment development [25, 26]. Notably, joint stiffness and cardiac involvement were confined to WMS1 patients, whereas the WMS4 patient exhibited intellectual disability, reinforcing emerging genotype-specific variability reported in recent series [6]. Additionally, the presence of heterotopic ossifications and brachydactyly with short, broad metacarpals in certain individuals raised strong suspicion for *GNAS*-related AHO, particularly when accompanied by elevated PTH levels. This observation is consistent with established disease mechanisms involving impaired Gsα signaling and end-organ hormone resistance [4]. Furthermore, five novel variants were identified in our study across genes implicated in acromelic dysplasias, expanding the molecular spectrum.

**Table 2:** Overview of key clinical and molecular features in the patient cohort with acromelic dysplasia subtypes

Feature	Geleophysic dysplasia (*ADAMTSL2*)	Acromicric dysplasia (*FBN1)*	Weill–Marchesani syndrome (*ADAMTS10/17*)	AHO (*GNAS*)
Short stature	+ (may improve)	+ (progressive)	+	+ (develops over time)
Acromelia/brachydactyly	+	+	+	+
Joint limitation	+ + (prominent)	+ (mild)	+ (variable; less in WMS4)	−
Tiptoe walking	+ (characteristic)	−	±	−
Pseudomuscular build	+	−	+ (especially WMS1)	−
Facial dysmorphism	+	+	±	−
Ophthalmologic involvement	Rare (ptosis)	−	+ + (spherophakia, ectopia lentis, glaucoma)	−
Cardiac involvement	+ (valvular, ASD, PS)	±	+ (mainly WMS1)	−
Intellectual disability	−	−	± (WMS4)	−
Bone age	Delayed	Usually normal	Delayed	Normal
Radiographic hand findings	Broad proximal phalanges, short tubular bones	Short tubular bones, metacarpal notching	Short tubular bones, short 5th middle phalanx	Short, broad metacarpals
Key diagnostic clues	**Tiptoe walking, broad phalanges, delayed bone age**	**Milder joint involvement**	**Mandatory ocular findings**	**Heterotopic ossification, PTH elevation**

*AHO* Albright hereditary osteodystrophy, *AS* aortic stenosis, *ASD* atrial septal defect, *MR* mitral regurgitation, *PS* pulmonary stenosis, *WMS* Weill–Marchesani syndrome

In conclusion, comparative evaluation of our cohort revealed both shared and disorder-specific patterns among acromelic dysplasia syndromes. While short stature was common, distinctive features—such as progressive joint stiffness and delayed bone age in geleophysic dysplasia, milder joint involvement in acromicric dysplasia, prominent ocular abnormalities in WMS, and heterotopic ossifications in AHO—facilitated clinical differentiation. These results underscore the importance of integrating clinical, radiologic, and molecular data for accurate diagnosis and individualized follow-up.

## Data Availability

No datasets were generated or analysed during the current study.

## Abbreviations

AHO: Albright hereditary osteodystrophy
F: Family
P: Patient
PTH: Parathyroid hormone
SDS: Standard deviation score
TB5: TGF-β-binding protein-like domain 5
TGF-β: Transforming growth factor beta
TSH: Thyroid-stimulating hormone
VUS: Variants of uncertain significance
WMS: Weill–Marchesani syndrome

## References

[1] Costantini A, Guasto A, Cormier-Daire V (2023) TGF-beta and BMP signaling pathways in skeletal dysplasia with short and tall stature. Annu Rev Genomics Hum Genet 24:225–253. 10.1146/annurev-genom-120922-094107 37624666 10.1146/annurev-genom-120922-094107

[2] Stanley S, Balic Z, Hubmacher D (2020) Acromelic dysplasias: how rare musculoskeletal disorders reveal biological functions of extracellular matrix proteins. Ann N Y Acad Sci 1490:57–76. 10.1111/nyas.14465 32880985 10.1111/nyas.14465 PMC7921208

[3] Unger S, Ferreira CR, Mortier GR, Ali H, Bertola DR, Calder A, Cohn DH, Cormier-Daire V, Girisha KM, Hall C, Krakow D, Makitie O, Mundlos S, Nishimura G, Robertson SP, Savarirayan R, Sillence D, Simon M, Sutton VR, Warman ML, Superti-Furga A (2023) Nosology of genetic skeletal disorders: 2023 revision. Am J Med Genet A 191:1164–1209. 10.1002/ajmg.a.63132 36779427 10.1002/ajmg.a.63132 PMC10081954

[4] Thiele S, Werner R, Grotzinger J, Brix B, Staedt P, Struve D, Reiz B, Farida J, Hiort O (2015) A positive genotype-phenotype correlation in a large cohort of patients with pseudohypoparathyroidism type Ia and Pseudo-pseudohypoparathyroidism and 33 newly identified mutations in the GNAS gene. Mol Genet Genomic Med 3:111–120. 10.1002/mgg3.117 25802881 10.1002/mgg3.117 PMC4367083

[5] Marzin P, Thierry B, Dancasius A, Cavau A, Michot C, Rondeau S, Baujat G, Phan G, Bonniere M, Le Bourgeois M, Khraiche D, Pejin Z, Bonnet D, Delacourt C, Cormier-Daire V (2021) Geleophysic and acromicric dysplasias: natural history, genotype-phenotype correlations, and management guidelines from 38 cases. Genet Med 23:331–340. 10.1038/s41436-020-00994-x 33082559 10.1038/s41436-020-00994-x

[6] Marzin P, Rondeau S, Alessandri JL, Dieterich K, le Goff C, Mahaut C, Mercier S, Michot C, Moldovan O, Miolo G, Rossi M, Van-Gils J, Francannet C, Robert MP, Jais JP, Huber C, Cormier-Daire V (2024) Weill-Marchesani syndrome: natural history and genotype-phenotype correlations from 18 news cases and review of literature. J Med Genet 61:109–116. 10.1136/jmg-2023-109288 37734846 10.1136/jmg-2023-109288

[7] Sahin S, Hiort O, Thiele S, Evliyaoglu O, Tuysuz B (2017) Follow-up findings in a Turkish girl with pseudohypoparathyroidism type Ia caused by a novel heterozygous mutation in the GNAS gene. J Clin Res Pediatr Endocrinol 9:74–79. 10.4274/jcrpe.3191 27425121 10.4274/jcrpe.3191 PMC5363169

[8] Richards S, Aziz N, Bale S, Bick D, Das S, Gastier-Foster J, Grody WW, Hegde M, Lyon E, Spector E, Voelkerding K, Rehm HL, Committee ALQA (2015) Standards and guidelines for the interpretation of sequence variants: a joint consensus recommendation of the American College of Medical Genetics and Genomics and the Association for Molecular Pathology. Genet Med 17:405–424. 10.1038/gim.2015.30 25741868 10.1038/gim.2015.30 PMC4544753

[9] Lee C-L, Chuang C-K, Chiu H-C, Chang Y-H, Tu Y-R, Lo Y-T, Wu J-Y, Lin H-Y, Lin S-P (2026) Case report: novel ADAMTSL2 compound heterozygous mutations in geleophysic dysplasia with bilateral glaucoma and keratoconus-like corneal ectasia. Front Genet 17. 10.3389/fgene.2026.1751809 10.3389/fgene.2026.1751809 PMC12883250 41664703

[10] Allali S, Le Goff C, Pressac-Diebold I, Pfennig G, Mahaut C, Dagoneau N, Alanay Y et al (2011) Molecular screening of ADAMTSL2 gene in 33 patients reveals the genetic heterogeneity of geleophysic dysplasia. J Med Genet 48:417–421. 10.1136/jmg.2010.087544 21415077 10.1136/jmg.2010.087544 PMC4413937

[11] Le Goff C, Mahaut C, Wang LW, Allali S, Abhyankar A, Jensen S, Zylberberg L et al (2011) Mutations in the TGFbeta binding-protein-like domain 5 of FBN1 are responsible for acromicric and geleophysic dysplasias. Am J Hum Genet 89:7–14. 10.1016/j.ajhg.2011.05.012 21683322 10.1016/j.ajhg.2011.05.012 PMC3135800

[12] Chen ZY, Cao Y, Yang J, He XH, Liu LP, Yuan YH (2025) Case report: a case of severe pulmonary hypertension combined with FBN1 mutation associated geleophysic dysplasia. Front Pediatr 13:1642390. 10.3389/fped.2025.1642390 40740820 10.3389/fped.2025.1642390 PMC12307399

[13] Sun C, Xu D, Pei Z, Yang L, Qiao Z, Lu W, Luo F, Qiu Z (2020) Separation in genetic pathogenesis of mutations in FBN1-TB5 region between autosomal dominant acromelic dysplasia and Marfan syndrome. Birth Defects Res 112:1834–1842. 10.1002/bdr2.1814 33030311 10.1002/bdr2.1814

[14] Guner Yilmaz B, Akgun-Dogan O, Ozdemir O, Yuksel B, Hatirnaz Ng O, Bilguvar K, Ay B et al (2024) Rapid genome sequencing for critically ill infants: an inaugural pilot study from Turkey. Front Pediatr 12:1412880. 10.3389/fped.2024.1412880 39026936 10.3389/fped.2024.1412880 PMC11254770

[15] Tian F, Dong X, Yuan R, Hou X, Qing J, Li Y (2024) Case report: two different acromelic dysplasia phenotypes in a Chinese family caused by a missense mutation in FBN1 and a literature review. Front Pediatr 12:1428513. 10.3389/fped.2024.1428513 39077065 10.3389/fped.2024.1428513 PMC11284092

[16] Shan YC, Yang ZC, Ma L, Ran N, Feng XY, Liu XM, Fu P, Yi MJ (2021) A review of three Chinese cases of acromicric/geleophysic dysplasia with FBN1 mutations. Int J Gen Med 14:1873–1880. 10.2147/IJGM.S306018 34040419 10.2147/IJGM.S306018 PMC8139683

[17] Piccolo P, Sabatino V, Mithbaokar P, Polishchuk E, Hicks J, Polishchuk R, Bacino CA, Brunetti-Pierri N (2019) Skin fibroblasts of patients with geleophysic dysplasia due to FBN1 mutations have lysosomal inclusions and losartan improves their microfibril deposition defect. Mol Genet Genomic Med 7:e844. 10.1002/mgg3.844 31350823 10.1002/mgg3.844 PMC6732269

[18] Verberne EA, Westermann JM, de Vries TI, Ecury-Goossen GM, Lo ANSM, Manshande ME, Faries S, Veenhuis HD, Philippi P, Falix FA, Rosina-Angelista I, Ponson-Wever M, Rafael-Croes L, Thorsen P, Arends E, de Vroomen M, Nagelkerke SQ, Tilanus M, van der Veken LT, Huijsdens-van Amsterdam K, van der Kevie-Kersemaekers AM, Alders M, Mannens M, van Haelst MM (2022) Genetic care in geographically isolated small island communities: 8 years of experience in the Dutch Caribbean. Am J Med Genet A 188:1777–1791. 10.1002/ajmg.a.62708 35253369 10.1002/ajmg.a.62708 PMC9314971

[19] Alankarage D, Enriquez A, Steiner RD, Raggio C, Higgins M, Milnes D, Humphreys DT, Duncan EL, Sparrow DB, Giampietro PF, Chapman G, Dunwoodie SL (2022) Myhre syndrome is caused by dominant-negative dysregulation of SMAD4 and other co-factors. Differentiation 128:1–12. 10.1016/j.diff.2022.09.002 36194927 10.1016/j.diff.2022.09.002 PMC10442510

[20] Alagia M, Cappuccio G, Pinelli M, Torella A, Brunetti-Pierri R, Simonelli F, Limongelli G, Oppido G, Nigro V, Brunetti-Pierri N, Tudp (2018) A child with Myhre syndrome presenting with corectopia and tetralogy of Fallot. Am J Med Genet A 176:426–430. 10.1002/ajmg.a.38560 10.1002/ajmg.a.38560 PMC5814867 29230941

[21] Catana A, Simonescu-Colan R, Cuzmici-Barabas Z, Militaru D, Iordanescu I, Militaru MS (2022) First documented case of Myhre syndrome in Romania: a case report. Exp Ther Med 23:323. 10.3892/etm.2022.11252 35386616 10.3892/etm.2022.11252 PMC8972842

[22] Yang DD, Rio M, Michot C, Boddaert N, Yacoub W, Garcelon N, Thierry B, Bonnet D, Rondeau S, Herve D, Guey S, Angoulvant F, Cormier-Daire V (2022) Natural history of Myhre syndrome. Orphanet J Rare Dis 17:304. 10.1186/s13023-022-02447-x 35907855 10.1186/s13023-022-02447-x PMC9338657

[23] Yang K, Wang X, Wang WQ, Han MY, Hu LM, Kang DY, Yang JY, Liu M, Gao X, Yuan YY, Xu JC (2023) A newborn male with Myhre syndrome, hearing loss, and complete syndactyly of fingers 3–4. Mol Genet Genomic Med 11:e2103. 10.1002/mgg3.2103 36373990 10.1002/mgg3.2103 PMC10009913

[24] Tuysuz B, Kasap B, Uludag Alkaya D, Alp Unkar Z, Koseoglu P, Geyik F, Ozer E, Onal H, Gezdirici A, Ercan O (2023) Investigation of (Epi)genetic causes in syndromic short children born small for gestational age. Eur J Med Genet 66:104854. 10.1016/j.ejmg.2023.104854 37758162 10.1016/j.ejmg.2023.104854

[25] Morales J, Al-Sharif L, Khalil DS, Shinwari JM, Bavi P, Al-Mahrouqi RA, Al-Rajhi A, Alkuraya FS, Meyer BF, Al Tassan N (2009) Homozygous mutations in ADAMTS10 and ADAMTS17 cause lenticular myopia, ectopia lentis, glaucoma, spherophakia, and short stature. Am J Hum Genet 85:558–568. 10.1016/j.ajhg.2009.09.011 19836009 10.1016/j.ajhg.2009.09.011 PMC2775842

[26] Mularczyk EJ, Singh M, Godwin ARF, Galli F, Humphreys N, Adamson AD, Mironov A, Cain SA, Sengle G, Boot-Handford RP, Cossu G, Kielty CM, Baldock C (2018) ADAMTS10-mediated tissue disruption in Weill-Marchesani syndrome. Hum Mol Genet 27:3675–3687. 10.1093/hmg/ddy276 30060141 10.1093/hmg/ddy276 PMC6196651

